# Supplementary material for: Defective oxygen inert phase stabilized high-voltage nickel-rich cathode for high-energy lithium-ion batteries
Source: Nat Commun. 2023 Dec 6;14:8087. doi: 10.1038/s41467-023-43792-0 (PMC10700321; doi:10.1038/s41467-023-43792-0)
Supplement: Supplementary file 1 — Supplementary Information [file 41467_2023_43792_MOESM1_ESM.pdf]

Supplementary Information

Defective oxygen inert phase stabilized high-voltage nickel-rich cathode for high-energy lithium-ion batteries

Zhongsheng Dai,[1] Zhujie Li,[2] Renjie Chen,[1,2,3*] Feng Wu,[1,2,3] Li Li[1,2,3*]

[1]Beijing Key Laboratory of Environmental Science and Engineering, School of Materials Science and Engineering, Beijing Institute of Technology, Beijing 100081, P. R. China

[2]Advanced Technology Research Institute, Beijing Institute of Technology, Jinan 250300, P. R. China

[3]Collaborative Innovation Center of Electric Vehicles in Beijing, Beijing 100081, P. R. China

*Corresponding author. E-mail: chenrj@bit.edu.cn; lily863@bit.edu.cn

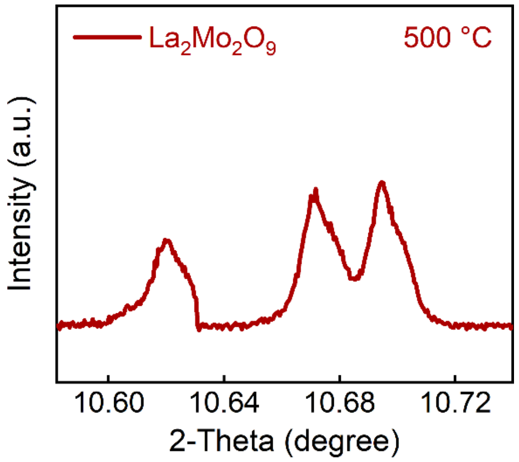

Supplementary Fig. 1. Selected XRD pattern for $La_2Mo_2O_9$ (LMO).

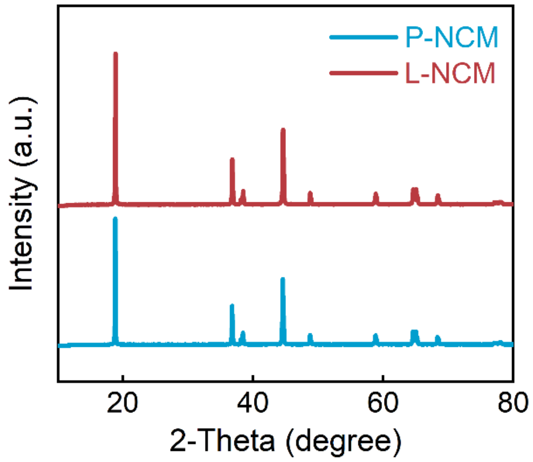

Supplementary Fig. 2. XRD patterns for uncycled P-NCM and L-NCM.

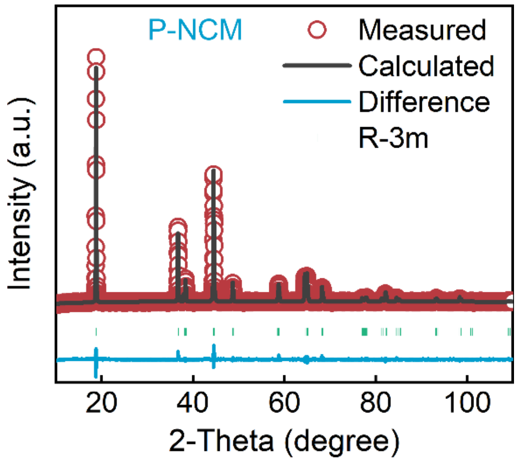

Supplementary Fig. 3. XRD refinement result for uncycled P-NCM.

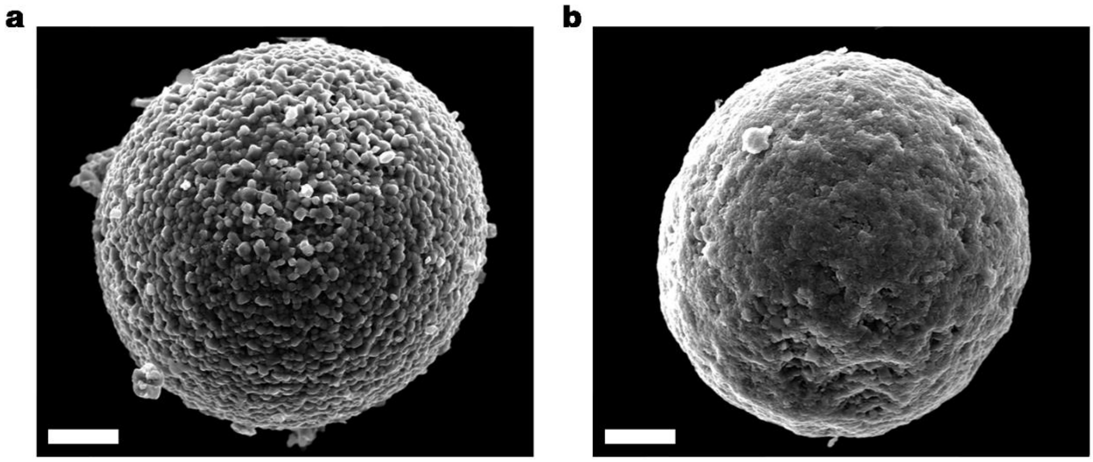

Supplementary Fig. 4. a, b SEM images for uncycled P-NCM (**a**) and L-NCM (**b**). Scale bars, 3 μm.

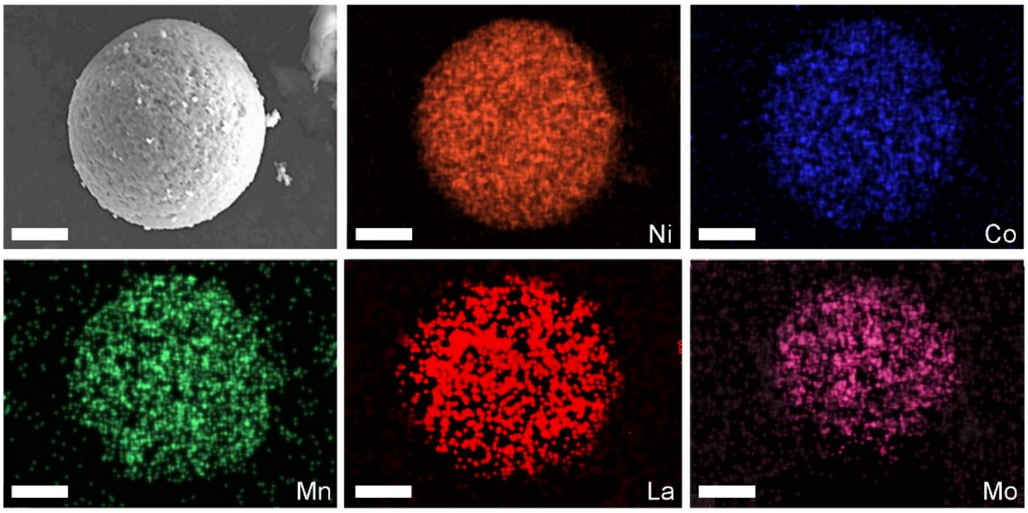

Supplementary Fig. 5. SEM and corresponding mapping images for uncycled L-NCM. Scale bars, 5 μm.

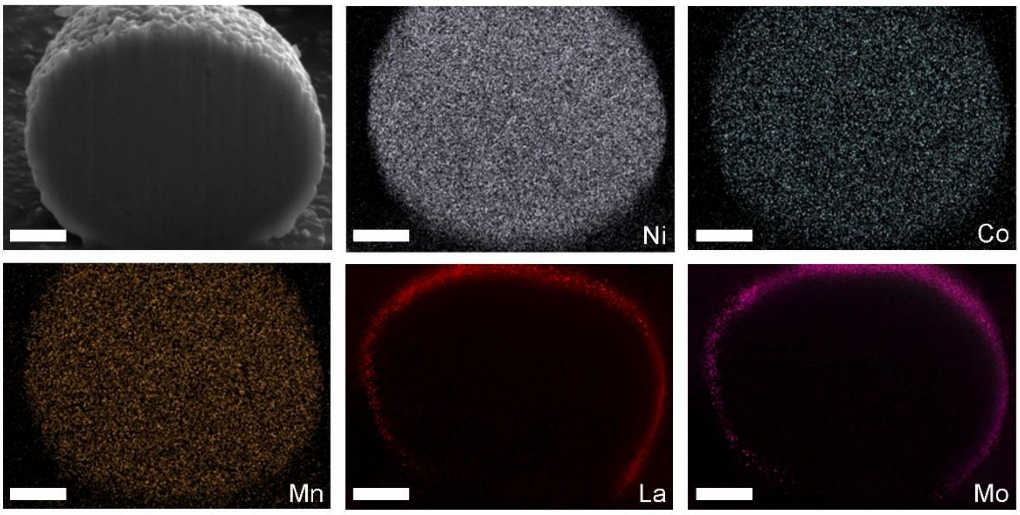

Supplementary Fig. 6. Cross-section morphology and the corresponding EDS mapping images for uncycled L-NCM. Scale bars, 3 μm.

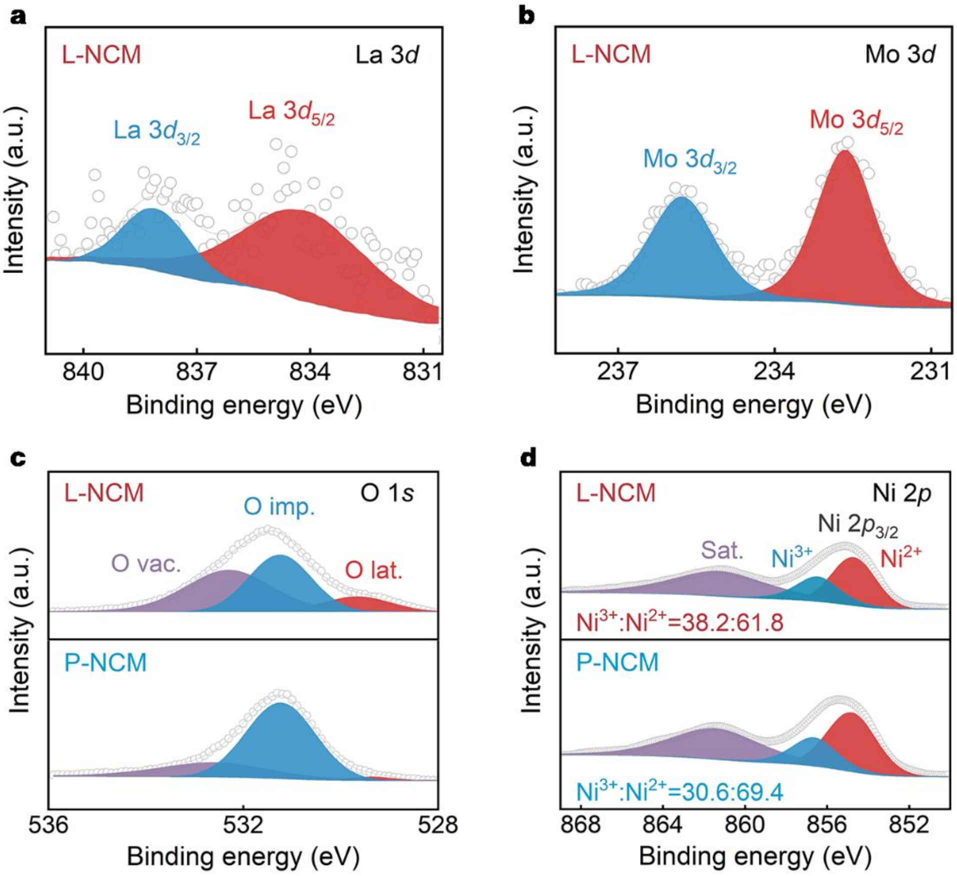

Supplementary Fig. 7. a-d XPS spectra of La 3*d* (**a**) and Mo 3*d* (**b**) for uncycled L-NCM, O 1*s* (**c**) and Ni 2*p* (**d**) for uncycled P-NCM and L-NCM. Note: O vac. is O vacancy, O imp. is O impurity, O lat. is O lattice, Sat. is satellite peak.

As shown in the fitted XPS spectra, the signals of La and Mo elements appeared in L-NCM surface (Supplementary Fig. 7a, b), which could be assigned to the 3*d* spin-orbit doublet[1,2], demonstrating the successful LMO deposition. Furthermore, the ratios of O vacancy located at 532.2 eV was obviously elevated after LMO modification (Supplementary Fig. 7c)[3], confirming an enriched oxygen vacancies interface were established in L-NCM surface. Moreover, the Ni 2*p* signals could be divided into two peaks at 854.7 and 856.5 eV (the purple is satellite peak, Supplementary Fig. 7d)[4], which were ascribed to $2p_{3/2}$ of Ni^{2+} and Ni^{3+} cations, respectively.

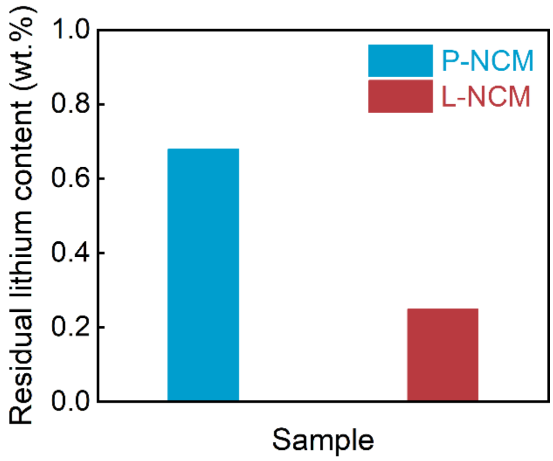

Supplementary Fig. 8. Residual lithium analysis for uncycled P-NCM and L-NCM.

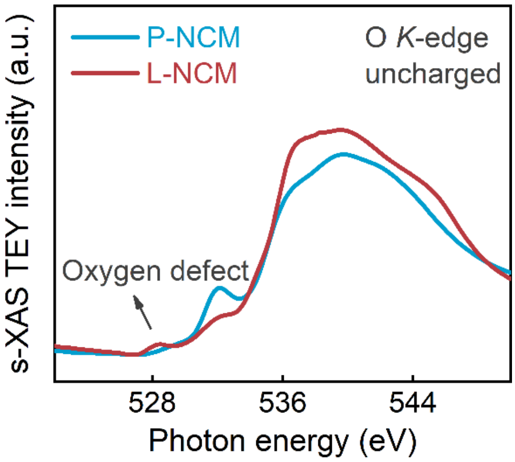

Supplementary Fig. 9. s-XAS spectra of O *K*-edge for uncharged P-NCM and L-NCM.

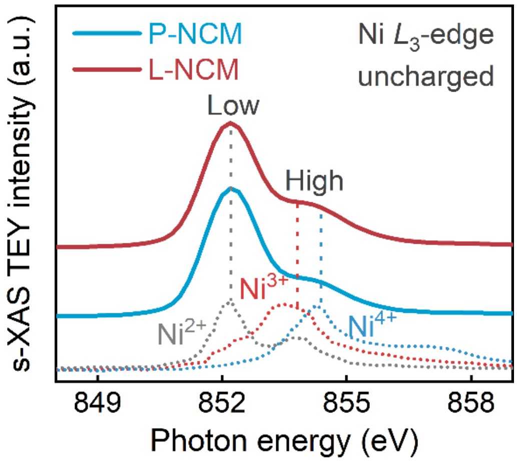

Supplementary Fig. 10. s-XAS spectra of Ni L_3-edge for uncharged P-NCM and L-NCM.

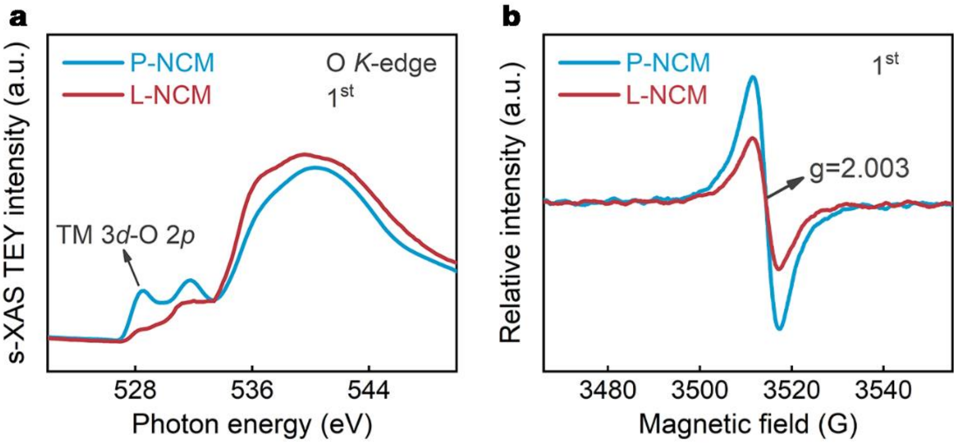

Supplementary Fig. 11. a s-XAS spectra of O *K*-edge at 1st charged state for P-NCM and L-NCM. **b** EPR profile at 1st charged state for P-NCM and L-NCM.

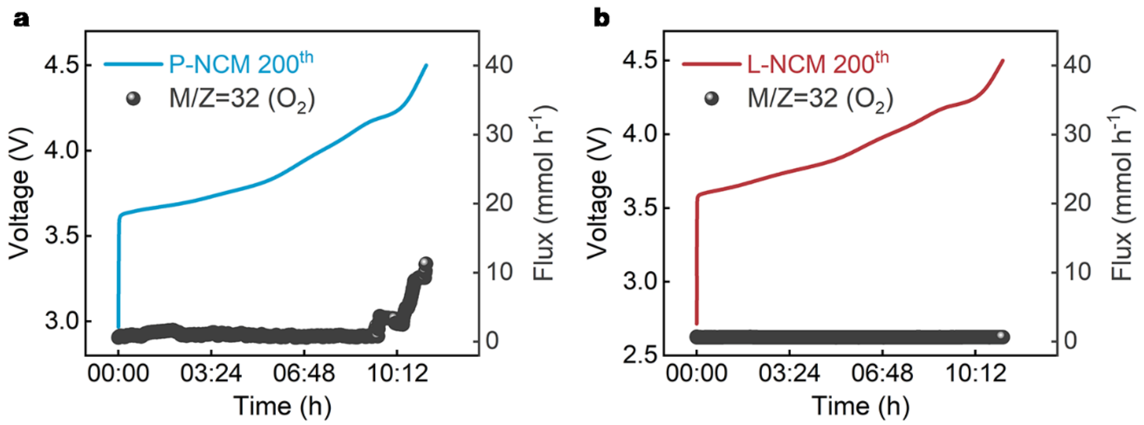

Supplementary Fig. 12. a, b In-situ DEMS data for P-NCM (**a**) and L-NCM (**b**) during 200th charge cycle under 2.7-4.5 V.

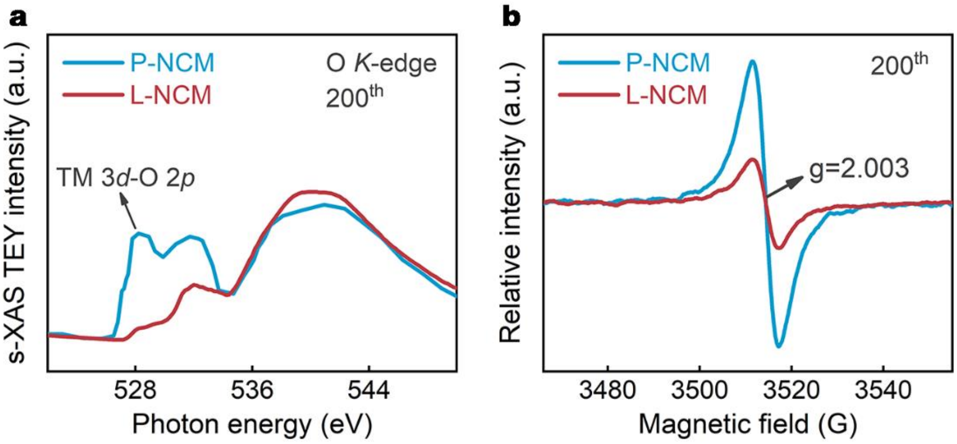

Supplementary Fig. 13. a s-XAS spectra of O *K*-edge at 200th charged state for P-NCM and L-NCM. **b** EPR profile at 200th charged state in P-NCM and L-NCM.

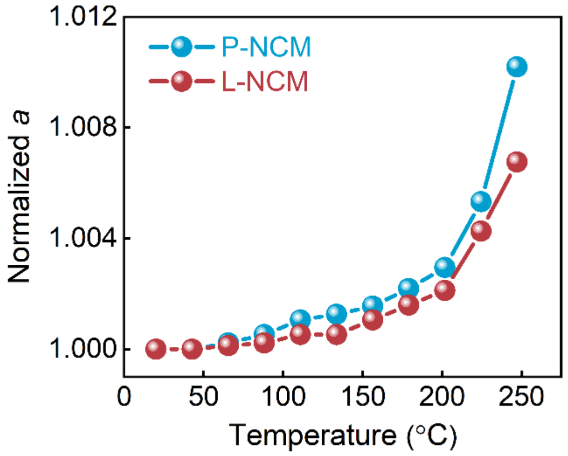

Supplementary Fig. 14. Evolutions of normalized *a*-axis for P-NCM and L-NCM.

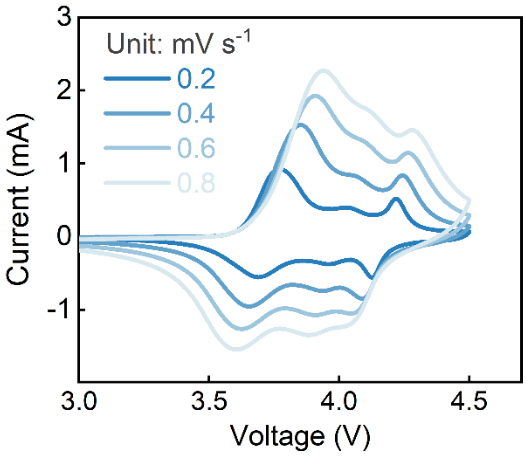

Supplementary Fig. 15. CV curves for P-NCM under different scan rates.

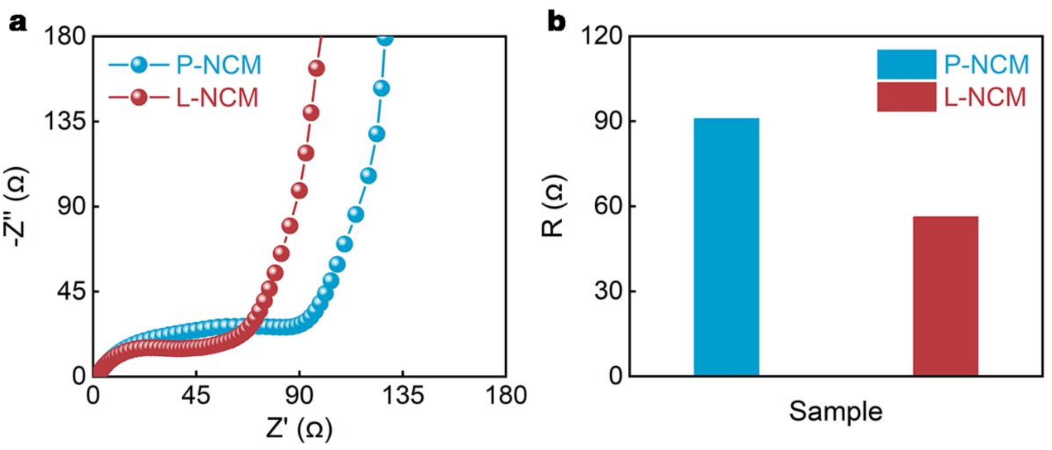

Supplementary Fig. 16. a Nyquist plots for P-NCM and L-NCM after 200 cycles. **b** R_{ct} values.

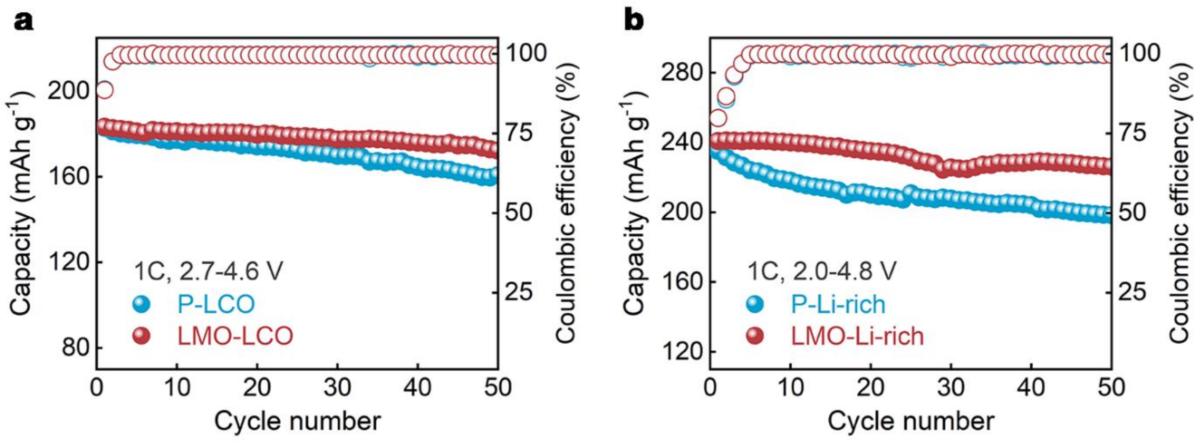

Supplementary Fig. 17. a Cycling performances for pristine $LiCoO_2$ (P-LCO) and LMO modified sample (LMO-LCO) in coin-type half cells at 1C under 2.7-4.6 V. **b** Cycling performances for pristine Li-rich (P-Li-rich) and LMO modified sample (LMO-Li-rich) in coin-type half cells at 1C under 2.0-4.8 V.

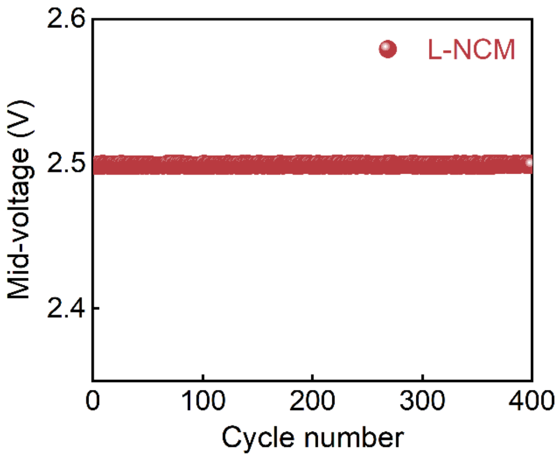

Supplementary Fig. 18. Voltage curve for L-NCM upon 400 cycles in pouch-type full cell at 0.5C under 2.7-4.2 V.

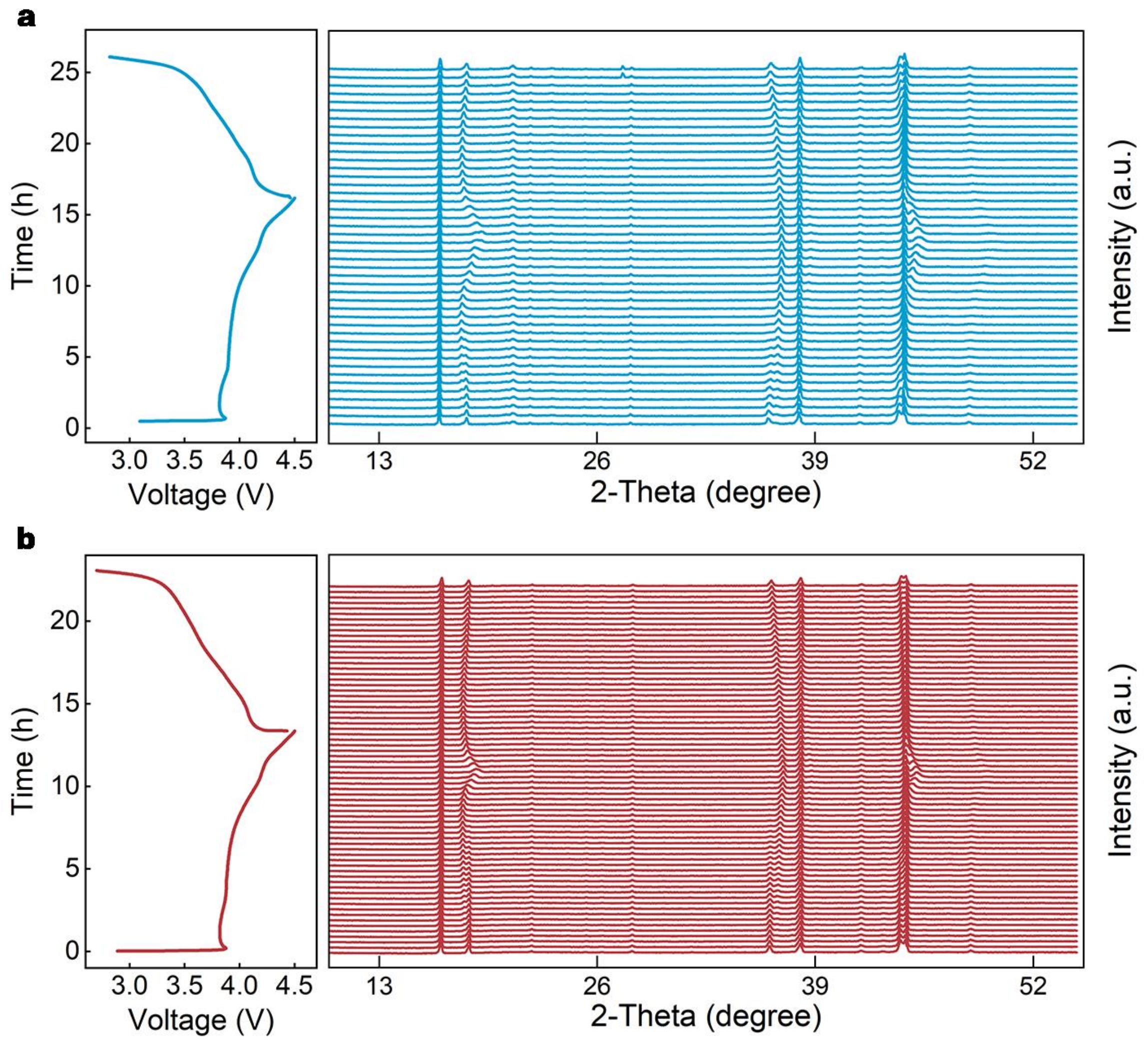

Supplementary Fig. 19. a, b Initial charge/discharge curves (left, 0.1C and 2.7-4.5 V) and in-situ XRD patterns (right) for P-NCM (**a**) and L-NCM (**b**).

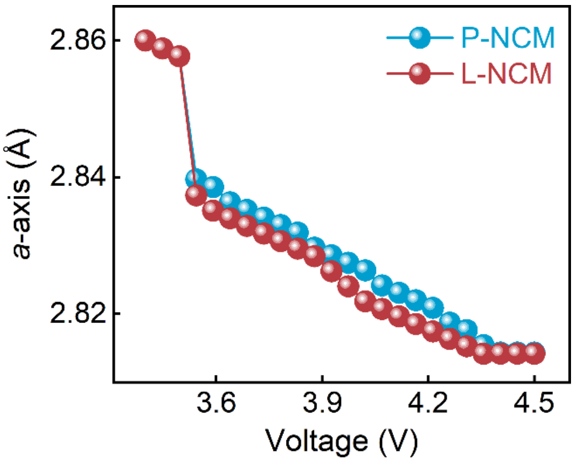

Supplementary Fig. 20. Lattice parameter *a* for P-NCM and L-NCM between 2.7 V and 4.5 V.

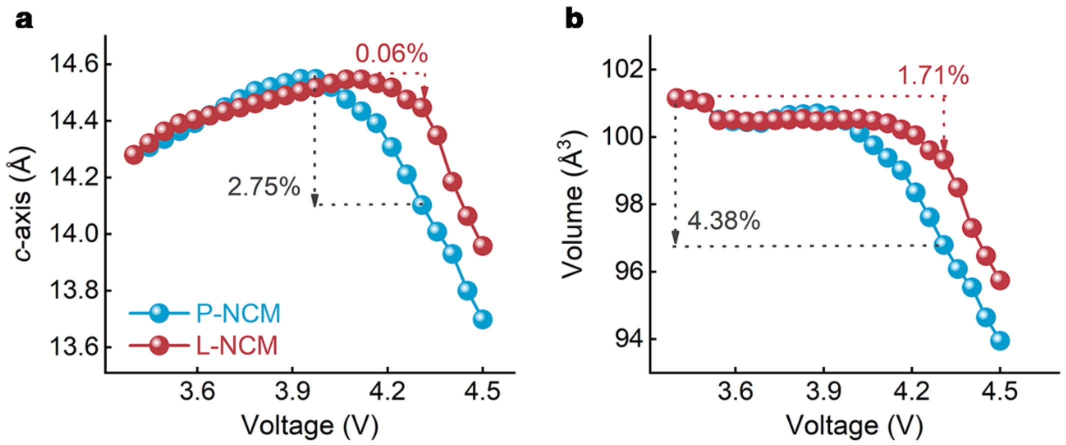

Supplementary Fig. 21. a, b Comparation of lattice parameters *c* (**a**) and volume (**b**) for P-NCM and L-NCM between 2.7 V and 4.3 V.

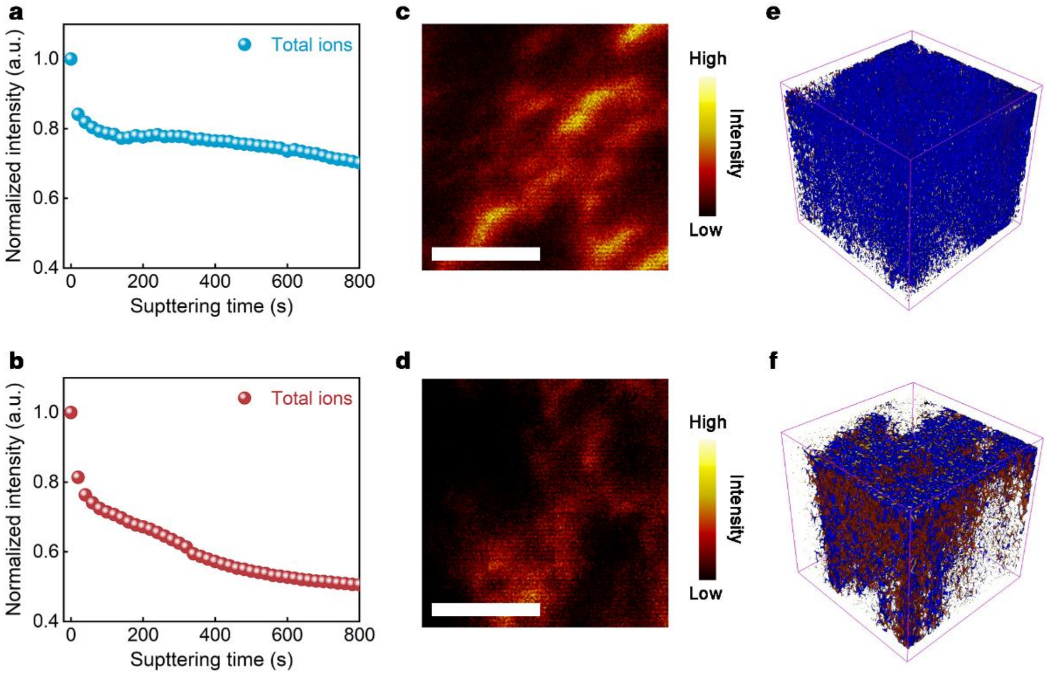

Supplementary Fig. 22. a, b Total ions distributions obtained by TOF-SIMS in P-NCM (**a**) and L-NCM (**b**) after 200 cycles. **c, d** Merged ions distributions of PO_2^-, C_2HO^-, NiF_3^- and MnF_3^- on P-NCM (**c**) and L-NCM (**d**) surface. Scale bars, 20 μm. **e, f** Merged depth ions distributions of PO_2^-, C_2HO^-, NiF_3^- and MnF_3^- on P-NCM (**e**) and L-NCM (**f**).

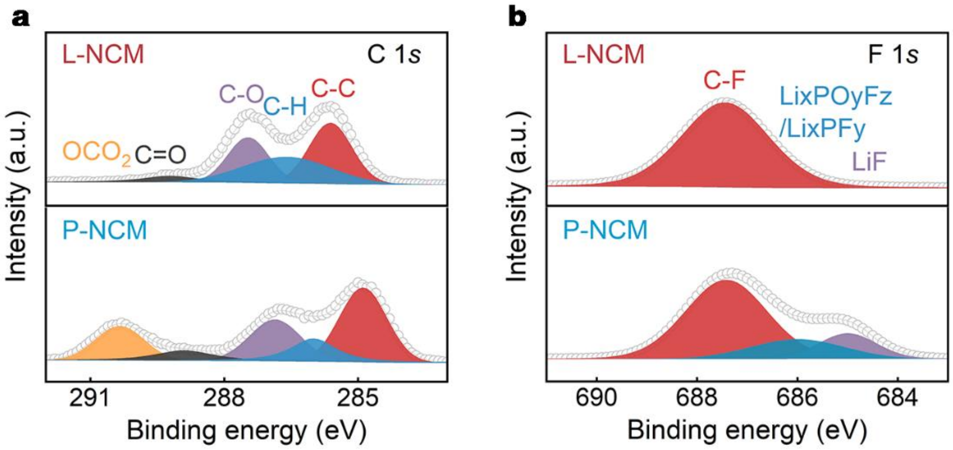

Supplementary Fig. 23. a, b XPS spectra for P-NCM and L-NCM cathodes after 200 cycles, C 1*s* (**a**) and F 1*s* (**b**).

As exhibited in C 1*s* peaks (Supplementary Fig. 23a), the C-C peaks at 285.1 eV originated from the Super P, while the peaks located at 286.2 eV (C-H) were related to PVDF binder[5]. The C-O (287.2 eV), C=O (289.1 eV) and OCO_2 (290.2 eV) species were attributed to the electrolyte decomposition[6], thus the increased C-O, C=O and OCO_2 intensity illustrated the aggravated electrolyte decomposition on P-NCM electrode surface. Additionally, as shown in Supplementary Fig. 23b, the LiF (684.3 eV) and LixPOyFz/LixPFy (685.2 eV) in F 1*s* peaks were derived from the parasitic reactions at the electrode/electrolyte interface and were identified as the components of cathode-electrolyte interface (CEI)[7,8]. The C-F (687.1 eV) bonds could be ascribed to the PVDF binder deposited on electrode surface. Therefore, the almost disappeared LixPOyFz/LixPFy and LiF peaks in L-NCM surface compared with P-NCM confirmed the mitigated electrolyte decomposition, coinciding with above-mentioned C 1*s* peaks analysis.

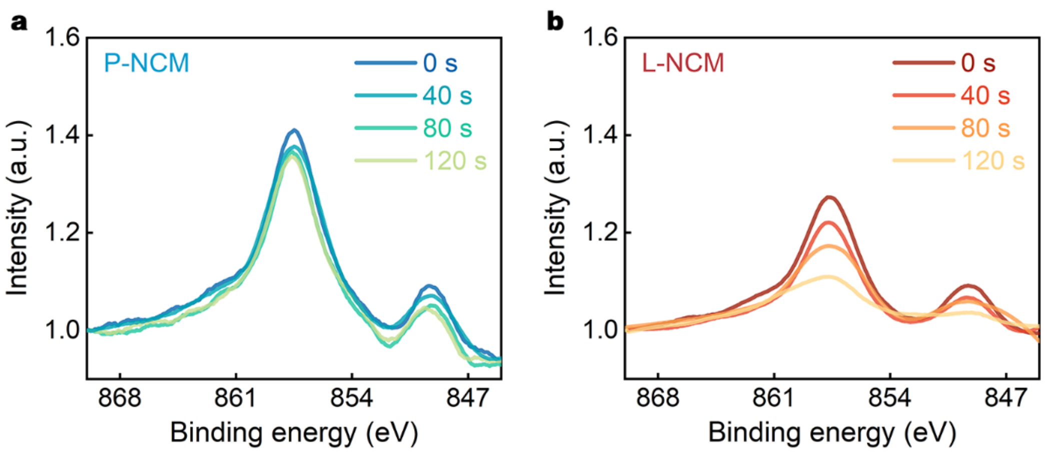

Supplementary Fig. 24. a, b In-depth XPS spectra for Ni on LMA surface after 200 cycles, coupled with P-NCM (**a**) and coupled with L-NCM (**b**).

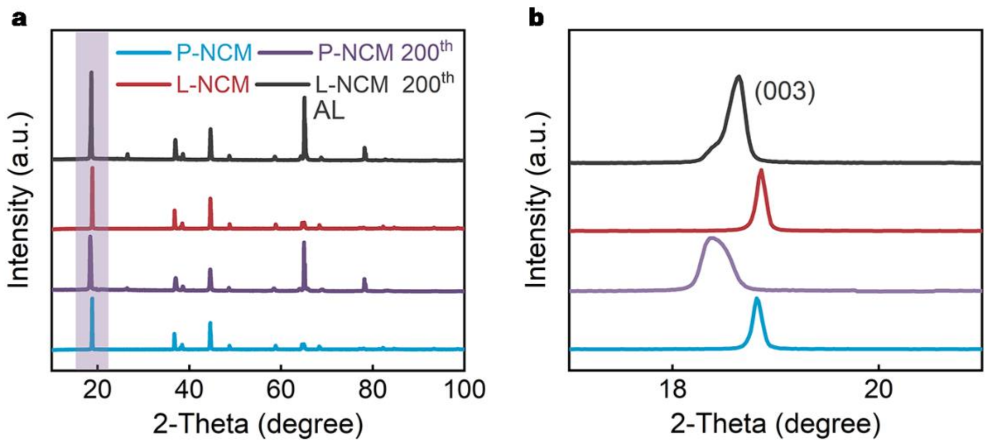

Supplementary Fig. 25. a, b XRD patterns (**a**) and magnified (003) peaks (**b**) for P-NCM and L-NCM before cycle and after 200 cycles.

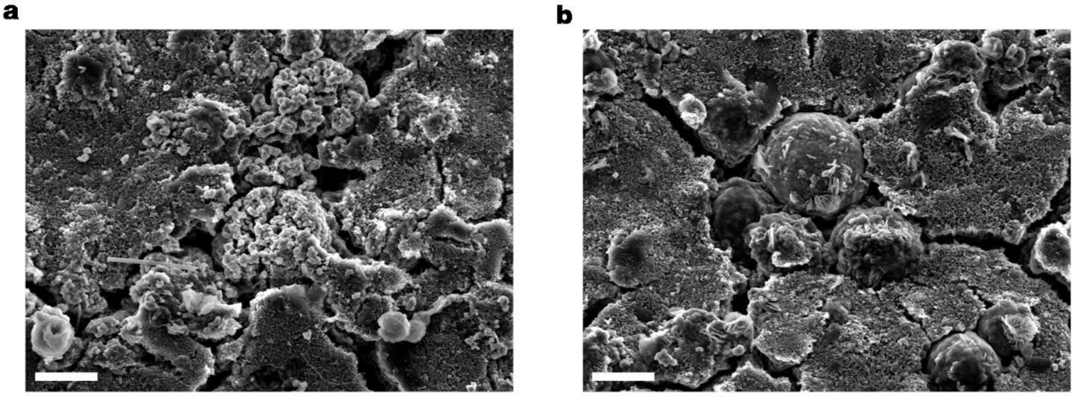

Supplementary Fig. 26. a, b SEM images for P-NCM (**a**) and L-NCM (**b**) after 200 cycles. Scale bars, 15 μm.

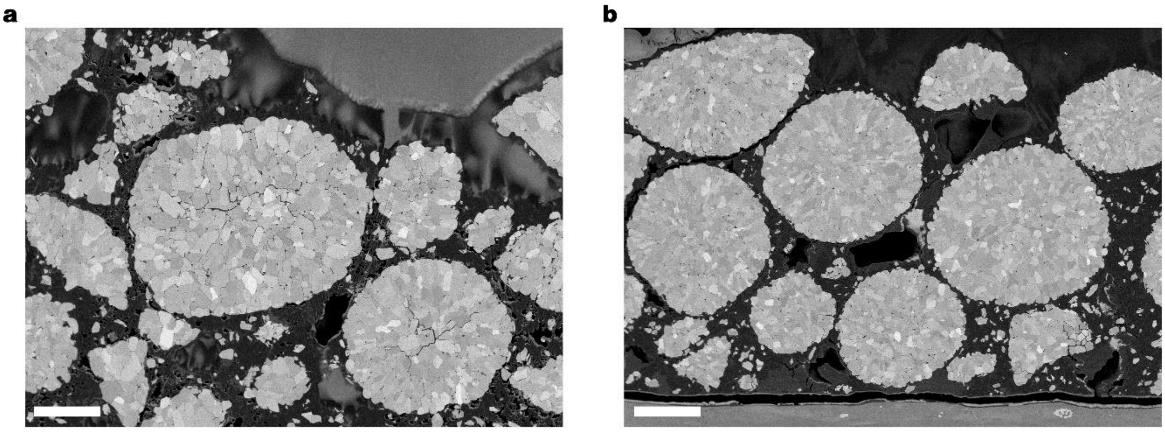

Supplementary Fig. 27. a, b Cross-section SEM images for P-NCM (**a**) and L-NCM (**b**) after 200 cycles. Scale bars, 5 μm.

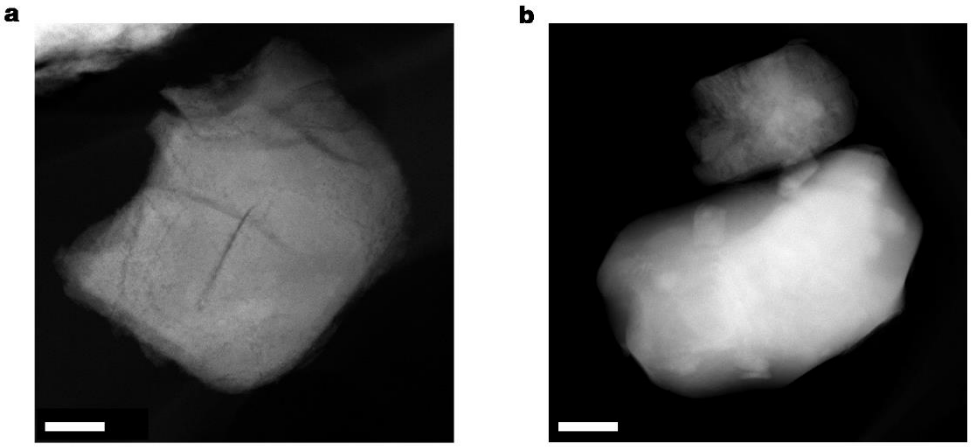

Supplementary Fig. 28. a, b HAADF-STEM images for P-NCM (**a**) and L-NCM (**b**) after 200 cycles. Scale bars, 100 nm.

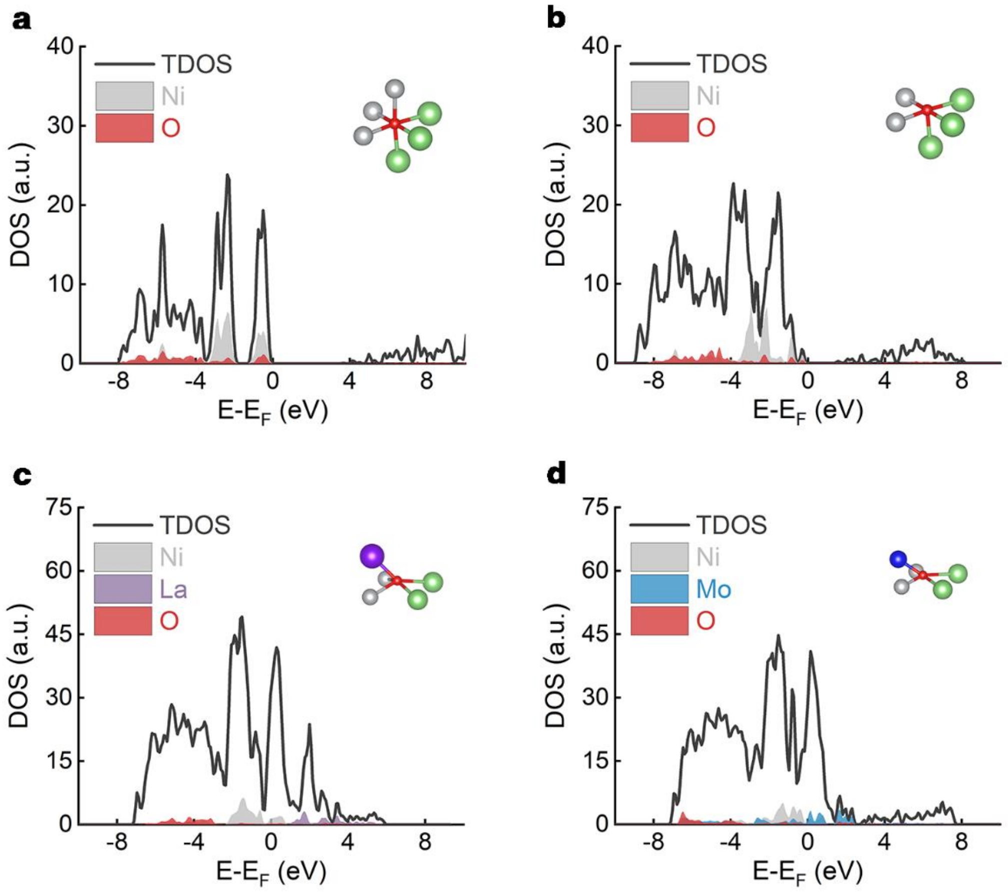

Supplementary Fig. 29. a-d Density of states for lattice oxygen coordinated by three Li and three Ni (**a**), surface oxygen coordinated by three Li and two Ni (**b**), surface oxygen coordinated by one La, two Li and two Ni (**c**) and surface oxygen coordinated by one Mo, two Li and two Ni (**d**). Note the elements: Li (green), Ni (gray), O (red), La (purple) and Mo (blue).

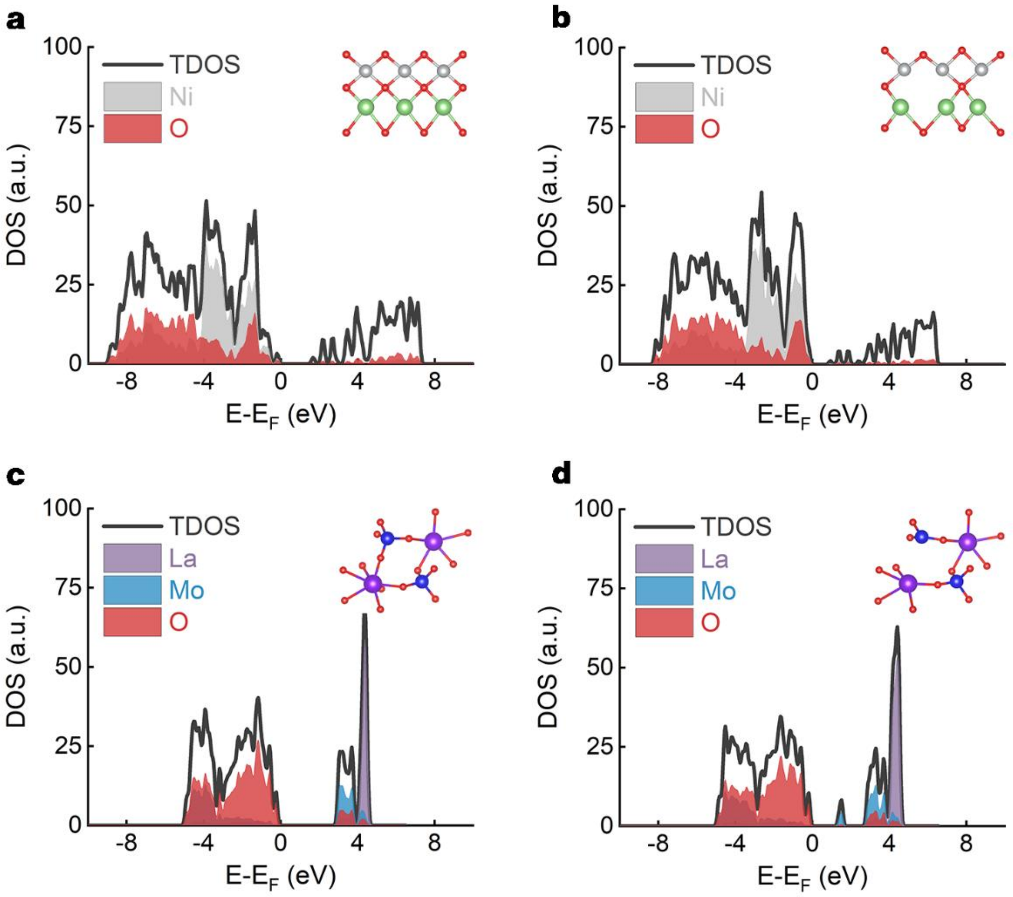

Supplementary Fig. 30. a-d Density of states for LNO (104) slab without defect (**a**) and with one oxygen defect (**b**), LMO (001) slab without defect (**c**) and with one oxygen defect (**d**). Note the elements: Li (green), Ni (gray), O (red), La (purple) and Mo (blue).

Supplementary Table 1. Lattice parameters quantified by XRD Rietveld refinement.

**Sample**	*a* = *b* (Å)	*c* (Å)	V (Å^3)	Z_{OX}	S_{TMO6} (Å)	W_{LiO6} (Å)	Li^+/Ni^{2+} (%)
**P-NCM**	2.8452	14.2035	101.2285	0.2603	2.0746	2.6599	1.36
**L-NCM**	2.8566	14.2039	101.2298	0.2598	2.0889	2.6457	1.32

TM slab thickness[9]: $S_{TMO6} = 2(1/3 - Z_{OX}) \times c$, Li slab thickness: W_{LiO6} (Å) = $(c/3) - S_{TMO6}$.

Supplementary Table 2. Lithium diffusion coefficient ($cm^2\ s^{-1}$) calculated by fitting CV data.

State	P-NCM	L-NCM
**Charge**	4.65×10^{-11}	8.68×10^{-11}
**Discharge**	2.31×10^{-11}	5.41×10^{-11}

Supplementary Table 3. Capacity retentions after 100 cycles under different voltages.

Sample	2.7-4.3 V	2.7-4.4 V	2.7-4.5 V
**P-NCM**	80.2%	65.4%	57.1%
**L-NCM**	93.9%	87.2%	84.8%

Supplementary Table 4. Comparison in electrochemical performances of L-NCM under different voltages with the recent similar-focused literatures.

Bulk material	Modification strategy	Voltage range and current density	Cycle number and capacity retention	Active material (mg cm^{-2})	Reference
$Li_{0.8}Ni_{0.1}Co_{0.1}Mn_{0.1}O_2$	$La_2Mo_2O_9$-coating	2.7-4.3 V, C/3	100, ~ 93.9%	~ 10.0	This work
$Li_{0.8}Ni_{0.1}Co_{0.1}Mn_{0.1}O_2$	$La_2Mo_2O_9$-coating	2.7-4.4 V, C/3	100, ~ 87.2%	~ 10.0	This work
$Li_{0.8}Ni_{0.1}Co_{0.1}Mn_{0.1}O_2$	$La_2Mo_2O_9$-coating	2.7-4.5 V, C/3	100, ~ 84.8%	~ 10.0	This work
$Li_{0.8}Ni_{0.1}Co_{0.1}Mn_{0.1}O_2$	DMS-electrolyte additive	2.75-4.5 V, 1C	100, ~ 73.5%	~ 4.3	10
$LiNi_{0.885}Co_{0.1}Al_{0.015}O_2$	B-doping	2.7-4.3 V, 0.5C	100, ~ 95.5%	~ 12.0	11
$Li_{0.8}Ni_{0.1}Co_{0.1}Mn_{0.1}O_2$	Sulfonamide-electrolyte additive	3.0-4.7 V, 0.5C	90, ~ 88%	~ 7.5	12
$Li_{0.8}Ni_{0.1}Co_{0.1}Mn_{0.1}O_2$	Co_xB-coating	3.0-4.4 V, 1C	100, ~ 87%	~ 10.5	13
$Li_{0.8}Ni_{0.1}Co_{0.1}Mn_{0.1}O_2$	Element gradient distribution	2.7-4.4 V, 0.5C	100, ~ 85.1%	~ 5.0	14
$Li_{0.6}Ni_{0.2}Co_{0.2}Mn_{0.2}O_2$	Annealing in steps	3.0-4.3 V, 1C	100, ~ 83%	~ 2.0	15
$Li_{0.6}Ni_{0.2}Co_{0.2}Mn_{0.2}O_2$	Single-crystal	3.0-4.3 V, 1C	100, ~ 89.1%	~ 2.0	16
$Li_{0.6}Ni_{0.2}Co_{0.2}Mn_{0.2}O_2$	Single-crystal	3.0-4.3 V, 0.1C	100, ~ 83.6%	~ 21.5	17
$LiNi_{0.91}Co_{0.09}O_2$	Ta-doping	2.7-4.3 V, 0.5C	100, ~ 75%	~ 4.0	18

Note: 1C = 220 mA g^{-1}

Supplementary Table 5. Bader charge analysis.

	$La@LiNiO_2$	$Mo@LiNiO_2$
**Bader charge analysis**	− 1.2743 eV	− 1.2107 eV

Supplementary Table 6. Relative parameters of pouch-type full cell.

**Full cell parameters**		
	Cathode	Anode
Sample	L-NCM	Graphite (Gr)
Composition	L-NCM : PVDF : CNT : SP = 95% : 3% : 1% : 1%	Gr : PVDF : CNT : SP = 95% : 3% : 1% : 1%
Electrode density (g cm^{-3})	3.00	1.60
Negative/Positive (N/P) ratio	1.12/1.00	
One-side loading level (mg cm^{-2})	20.00	12.48
One-side capacity (mAg cm^{-2})	3.90	4.37
Initial charge capacity (mAh g^{-1})	217.00	384.60
Initial discharge capacity (mAh g^{-1})	195.00	350.00
Initial coulombic efficiency (%)	90.00	91.00
Voltage range (V)	2.7-4.2	

References

1 Zhu, X. et al. Epitaxial growth of an atom-thin layer on a $LiNi_{0.5}Mn_{1.5}O_4$ cathode for stable Li-ion battery cycling. *Nat. Commun.* **13**, 1565 (2022).

2 Geon, P. et al. Introducing high-valence elements into cobalt-free layered cathodes for practical lithium-ion batteries. *Nat. Energy* **7**, 946-954 (2022).

3 Wang, Q. et al. Unlocking anionic redox activity in O3-type sodium 3*d* layered oxides via Li substitution. *Nat. Mater.* **20**, 353-361 (2021).

4 Dai, Z. et al. Surface coupling between mechanical and electric fields empowering Ni-rich cathodes with superior cyclabilities for lithium-ion batteries. *Adv. Sci.* **9**, e2200622 (2022).

5 Bernardine, R. et al. Two electrolyte decomposition pathways at nickel-rich cathode surfaces in lithium-ion batteries. *Energy Environ. Sci.* **15**, 3416 (2022).

6 Zou, Y. et al. Interfacial model deciphering high-voltage electrolytes for high energy density, high safety, and fast-charging lithium-ion batteries. *Adv. Mater.* **33**, 2102964 (2021).

7 Yu, H. et al. Surface enrichment and diffusion enabling gradient-doping and coating of Ni-

rich cathode toward Li-ion batteries. *Nat. Commun.* **12**, 4564 (2021).

8 Xu, M. et al. Development of novel lithium borate additives for designed surface modification of high voltage $LiNi_{0.5}Mn_{1.5}O_4$ cathodes. *Energy Environ. Sci.* **9**, 1308 (2016).

9 Lee, W. et al. New insight into Ni-rich layered structure for next-generation Li rechargeable batteries. *Adv. Energy Mater.* **8**, 1701788 (2018).

10 Wu, F. et al. High-voltage and high-safety nickel-rich layered cathode enabled by a self-reconstructive cathode/electrolyte interphase layer. *Energy Storage. Mater.* **41**, 495 (2021).

11 Ryu, H. et al. A highly stabilized Ni-rich NCA cathode for high-energy lithium-ion batteries. *Mater. Today* **36**, 73 (2020).

12 Xue, W. et al. Ultra-high-voltage Ni-rich layered cathodes in practical Li metal batteries enabled by a sulfonamide-based electrolyte. *Nat. Energy* **6**, 495 (2021).

13 Ryu, H. et al. Reactive boride infusion stabilizes Ni-rich cathodes for lithium-ion batteries. *Nat. Energy* **6**, 362 (2022).

14 Lin, R. et al. Hierarchical nickel valence gradient stabilizes high-nickel content layered cathode materials. *Nat. Commun.* **12**, 2350 (2021).

15 Park, H. et al. In situ multiscale probing of the synthesis of a Ni-rich layered oxide cathode reveals reaction heterogeneity driven by competing kinetic pathways. *Nat. Chem.* **14**, 614 (2022).

16 Zhang, F. et al. Surface regulation enables high stability of single-crystal lithium-ion cathodes at high voltage. *Nat. Commun.* **11**, 3050 (2020).

17 Bi, Y. et al. Reversible planar gliding and microcracking in a single-crystalline Ni-rich cathode. *Science* **370**, 1313 (2020).

18 Sun, H. et al. Transition metal-doped Ni-rich layered cathode materials for durable Li-ion batteries. *Nat. Commun.* **12**, 6552 (2021).
